# On the Use of Laser Fragmentation for the Synthesis of Ligand-Free Ultra-Small Iron Nanoparticles in Various Liquid Environments

**DOI:** 10.3390/nano11061538

**Published:** 2021-06-10

**Authors:** Ondřej Havelka, Martin Cvek, Michal Urbánek, Dariusz Łukowiec, Darina Jašíková, Michal Kotek, Miroslav Černík, Vincenzo Amendola, Rafael Torres-Mendieta

**Affiliations:** 1 Institute for Nanomaterials, Advanced Technologies and Innovation, Technical University of Liberec, Studentská 1402/2, 461 17 Liberec, Czech Republic; ondrej.havelka@tul.cz (O.H.); darina.jasikova@tul.cz (D.J.); michal.kotek@tul.cz (M.K.); miroslav.cernik@tul.cz (M.Č.); 2 Centre of Polymer Systems, University Institute, Tomas Bata University in Zlín, Třída T. Bati 5678, 760 01 Zlín, Czech Republic; cvek@utb.cz (M.C.); murbanek@utb.cz (M.U.); 3 Faculty of Mechanical Engineering, Silesian University of Technology, Konarskiego 18 a St., 44-100 Gliwice, Poland; dariusz.lukowiec@polsl.pl; 4 Department of Chemical Sciences, University of Padova, via Marzolo 1, I-35131 Padova, Italy; vincenzo.amendola@unipd.it

**Keywords:** iron nanoparticles, ultra-small nanoparticles, nZVI, stabilization effect, laser fragmentation in liquid

## Abstract

Traditionally, the synthesis of nanomaterials in the ultra-small size regime (1–3 nm diameter) has been linked with the employment of excessive amounts of hazardous chemicals, inevitably leading to significant environmentally detrimental effects. In the current work, we demonstrate the potential of laser fragmentation in liquids (LFL) to produce highly pure and stable iron ultra-small nanoparticles. This is carried out by reducing the size of carbonyl iron microparticles dispersed in various polar solvents (water, ethanol, ethylene glycol, polyethylene glycol 400) and liquid nitrogen. The explored method enables the fabrication of ligand-free iron oxide ultra-small nanoparticles with diameter in the 1–3 nm range, a tight size distribution, and excellent hydrodynamic stability (zeta potential > 50 mV). The generated particles can be found in different forms, including separated ultra-small NPs, ultra-small NPs forming agglomerates, and ultra-small NPs together with zero-valent iron, iron carbide, or iron oxide NPs embedded in matrices, depending on the employed solvent and their dipolar moment. The LFL technique, aside from avoiding chemical waste generation, does not require any additional chemical agent, other than the precursor microparticles immersed in the corresponding solvent. In contrast to their widely exploited chemically synthesized counterparts, the lack of additives and chemical residuals may be of fundamental interest in sectors requiring colloidal stability and the largest possible number of chemically active sites, making the presented pathway a promising alternative for the clean design of new-generation nanomaterials.

Nanotechnology can provide answers to current and future issues facing humankind [1]. Since its discovery, it has become evident that precise control over material dimensions could lead to *ad-hoc* design solutions to nature’s most challenging problems. However, the central issue in the field is the impossibility of producing two elements with identical size. Given this problem, the scientific society has turned the tide by focusing on the smallest possible nanomaterial building blocks, in order to approach nanostructure creation with truly identical dimensions. This answer, known as ultra-small nanoparticles (NPs with dimensions of <3 nm) at present [2], not only facilitates the construction of highly precise materials, but can also lead to the discovery of new properties related to their dramatic surface-to-volume ratio. Some of their remarkable properties that have found application in many sectors are the appearance of fluorescence in noble metal ultra-small NPs [3], chirality [4], enhancement of chemical reactivity [5,6], modification of magnetic and electrochemical properties (with respect to their NP counterparts) [7,8], and increased thermal stability [9].

Among the currently explored elements for the design of ultra-small NPs, iron compounds have attracted a great deal of attention, due to their magnetic and electrochemical properties, abundance, and lower toxicity, compared to other magnetic options such as Co or Ni [10]. Such attributes have enabled their use in cell-labeling [11], drug delivery [12], theranostics [13], T${}_{1}$ contrast agents in MRI [14,15], magnetic hyperthermia [16], photothermal therapy [17], catalysis [18], and recently in magnetorheology [19].

The synthesis of Fe-based ultra-small NPs is mainly achieved by chemical vapor decomposition, micro-emulsion, controlled chemical co-precipitation, and thermal decomposition [20]. The most substantial issues related to these techniques are the production of large amounts of chemical waste materials, the usage of hazardous chemicals, and NP surface passivation using large amounts of stabilizing agents. In recent years, pulsed laser irradiation-based strategies have gained attention, due to their ability to produce nanostructures with novel properties and less environmental impact. For instance, Vitta et al. [21] have irradiated a pure Fe plate to produce stable α-Fe NPs with a predominant size of 5–45 nm. In their research, the metal target was submerged in an aqueous solution of sodium dodecyl sulfate (SDS), in order to ensure the stability of the produced NPs through micelle formation. Later, Amendola et al. [22] obtained Fe-based nanostructures after irradiating bulk iron in different organic solvents, including tetrahydrofuran, acetonitrile, dimethylformamide (DMF), dimethylsulfoxide, toluene, and ethanol, demonstrating that the chemistry of the solvent plays a significant role in determining the physicochemical properties of the final product. Other authors [23] have fabricated high-purity amorphous Fe NPs with sizes of 1–3 nm from a tiny iron wire in a low-pressure environment. They also found that the nature of the prepared NPs is determined by the target size, rather than the processing parameters.

Pulsed laser ablation has allowed for the formation of 50–110 nm magnetic α-Fe${}_{2}$O${}_{3}$ NPs after irradiating iron pellets immersed into DMF and SDS solutions [24]. These NPs have shown remarkable antibacterial activity against certain Gram-positive and Gram-negative bacteria. Recently, the number of liquids used for laser ablation has been expanded. De Bonis et al. [25] used distilled water and acetone to produce Fe-based nanostructures using laser-assisted methodologies. Fazio et al. [26] further extended the topic: aside from water, they used aqueous polyvinyl alcohol solution to produce Fe${}_{2}$O${}_{3}$ under various ablation parameters. The ablation parameters have been further correlated with the antimicrobial activity of their NPs and, most recently, Lahoz et al. [27] used distilled water and ethanol to produce colloidal dispersions of nanoscale zero-valent iron (nZVI) particles for biomedical and environmental applications. Furthermore, the control of the used solvent has also resulted in the oxidation control of metals; their alloying with metal salts dispersed in them, as has been demonstrated by Hu et al. [28,29]; or even in their graphitic coating, as has recently been shown by Davari et al. [30].

Given the current literature, it is evident that further research is necessary to explore the formation of iron-based NPs from different target materials after employing a larger variety of liquid environments, which is the main objective of the current work. Thus, we tackle the fundamental drawbacks of conventional chemical production methods by employing a technique known as laser fragmentation in liquids (LFL), in order to synthesize ligand-free Fe ultra-small NPs while avoiding chemical waste production. Unlike other laser-driven methods, the LFL strategy—which adheres to green chemistry principles [31]—enables precise control in the 1–5 nm size regime, making it perfect for the production of ultra-small NPs. For instance, Jendrzej S. et al. [32] demonstrated the potential of LFL for producing Pd, Pt, and Au NPs with sizes in the ultra-small regime. Waag F. et al. [33] also demonstrated its employability to obtain cobalt ferrite NPs with sizes up to 2 nm for their use in catalysis. Very recently, Yu M. et al. [34] obtained cobalt oxide sub-5 nm particles for the electrochemical oxygen evolution reaction.

Aside from a few reports, such as that of Ziefuss et al. [35], who investigated the influence of various ions on the size control over Au NPs when using LFL, there is still a lack of knowledge on a variety of liquid environments allowing for the production of ligand-free iron NPs with sizes in the ultra-small regime. Thus, the current work seeks to address this knowledge gap, through elucidating the effects of solvent polarity and type on the composition and colloidal stability of these materials.

In brief, LFL consists of focusing a pulsed laser beam into a colloid composed of micro- or nano-particles immersed in a liquid medium, in order to reduce the particle size. The size reduction is prompted by photothermal vaporization or Coulomb explosion (non-thermal). Both mechanisms can occur for any pulse duration (ns, ps, and fs), but it is more probable to observe the thermal mechanism when using ns pulses and the non-thermal mechanism when using ps or fs pulses. It should be mentioned that the usage of a pulse duration below the usual electron–phonon coupling time in NPs (∼ps), short wavelengths, extensive irradiation times, and massive fluence values can potentially help to scale up the process towards its industrialization. In the current work, we explore another scaling-up alternative, using a high repetition (kHz) ns pulsed laser to maximize the photothermal vaporization mechanism. In this process, the energy absorbed by the particles is distributed through their whole electronic lattice, promoting their heating, melting, and further surface evaporation. During this process, some energy is also transferred to the surrounding liquid medium, enabling the nearby liquid to reach its spinodal temperature, which promotes the formation of a cavitation bubble around the energy-transferring particle [36]. During the bubble’s lifetime (∼ns), the vapor detached from the particle’s surface starts to condense in the bubble, thus forming smaller NPs. The newly created particles interact with the bubble’s inner molecules and, depending on their nature, these interactions can lead to defects in the new NPs, chemical reduction, oxidation, or phase transformation. After the bubble’s collapse, the newly created NPs are released into the liquid medium [37]. As has been widely reported, the employment of polar solvents can enable the hydrodynamic stability of incoming products without the need for stabilization agents, by forming an electrical double layer around the NPs due to their electrostatic interaction with the liquid’s polar molecules [38]. The lack of ligands covering the surface of Fe-based ultra-small NPs is very attractive, especially for applications requiring further functionalization, such as biomedical or catalytic processes. Therefore, in an effort to find a suitable polar liquid enabling the best stabilization of the fabricated nanomaterial, we selected five solvents (ethanol, water, ethylene glycol, polyethylene glycol 400, and liquid nitrogen) with different dipolar moments (1.69 D, 1.85 D, 2.75 D, 3.70 D, and 0 D, respectively) to produce the precursor colloids for LFL. The samples consisted of 10 mL of each solvent and 0.4 mg/mL of carbonyl iron microparticles (HQ grade, BASF, Germany) with a nominal size of 2 μm and spherical shape. A MIX ARGOlab Vortex Mixer was used to mix the samples for 1 min. Note that higher concentrations would permit faster production, but massive precursor concentrations should also be accompanied by experimental strategies ensuring equal energy delivery to all the irradiated solids.

After preparation of the colloids, the samples (labeled according to the dispersing solvent) were irradiated by a Nd:YLF laser (Litron Lasers; LDY300 PIV Series), which has two optical cavities, delivers pulses of 150 ns full width at half maximum (FWHM) at a 527 nm central wavelength, with a 1 kHz repetition rate for each cavity. The laser fluence used to irradiate all the samples was 4.3 · 10${}^{4}$ J/cm${}^{2}$ (fluence calculation can be found in the Supplementary Material). According to the heating–melting–evaporation model [39], the selected fluence value should surpass the full evaporation threshold of iron microparticles (23 J/cm${}^{2}$) by three orders of magnitude (the details of the threshold value’s calculation can be found in the Supplementary Material). This high energy value selection ensures the size-reduction of microparticles without worrying about possible energy losses through the laser beam pathway. The irradiation took place according to the experimental architecture displayed in Figure 1.

In brief, a Galilean telescope (plano-concave lens, f = −150 mm; and plano-convex lens, f = 75 mm) was employed to magnify the incoming laser beam two times, reaching a measured diameter of 1.2 cm. A plano-convex lens (f = 35 mm) was subsequently employed, in order to focus the laser beam with a focal spot size of about 3.2 μm into the samples at a distance of 5 mm from the air–liquid interface, which had an optimal tolerance to maintain the focal point always inside the liquid solution during the LFL procedure, despite possible changes in the interface level due to continious stirring by a laboratory spatula at 100 rpm. The irradiation process lasted for 30 min and, after it finished, the samples were centrifuged for 15 min at 14,500 rpm and subsequently divided into supernatant (ultra-small NPs) and sediment (non-irradiated microparticles or particles irradiated with not enough energy to yield ultra-small NPs). A different approach was followed for the sample with liquid nitrogen. The sample was left to dry after irradiation; subsequently, the solid material was dispersed in ethanol for further centrifuging and characterization. Ethanol was selected, due to its lowest dipolar moment (except for liquid nitrogen) among the liquids used for LFL. Despite the fact that ethanol and liquid nitrogen evaporated during the irradiation process, these solvents were continuously supplemented throughout the experiment, keeping the same concentration and irradiated volume for the entire experiment’s duration, in order to avoid substantial modification of the processing conditions. Besides, as the laser beam was focused on the center of the container, it is possible to consider that the fragmentation process took place at a cryogenic or nearly cryogenic temperature.

The evaluation of the hydrodynamic size and stability of both supernatant and sediment re-dispersed in the corresponding pure solvent was performed by dynamic light scattering (DLS), using a Zetasizer Nano ZS90 (Malvern Instruments Ltd., United Kingdom laser wavelength centered at 632.8 nm, and detector placed at a scattering angle of 173${}^{\circ }$); the same instrument was used to evaluate the zeta potential of the samples, through laser Doppler electrophoresis (LDE). The precise size and morphology of the nanoparticles were evaluated by transmission electron microscopy (TEM), employing a microscope (JEM 2100, JEOL, Japan) using a bright LaB6 source operated at an acceleration voltage of 200 kV. The sample’s chemical composition was confirmed by energy-dispersive X-ray spectroscopy (EDX) linear scanning, using a detector (X-Max 80, Oxford Instruments, United Kingdom) incorporated in the scanning TEM microscope (S/TEM TITAN 80–300, FEI, United States) operated at an acceleration voltage of 100 kV. The same instrument was used to determine the crystallographic composition by selected area electron diffraction (SAED), using a high-angle annular dark-field detector. The surface nature of the ultra-small NPs was analyzed by X-ray photoelectron spectroscopy (XPS), using a spectrometer (Axis Supra, Kratos Analytical, United Kingdom) with an Al Kα X-ray source and a pass energy of 20 eV. The final ultra-small NPs concentration was assessed through Inductively coupled plasma optical emission spectrometry (ICP-OES), using a spectrometer (Optima 2100Dv, Perkin Elmer, United Kingdom) which reached a determination of trace elements with a detection limit of >0.2 ppb.

The hydrodynamic size distribution of particles before laser irradiation was centered at 1182 nm with an FWHM of 1093 nm for all solvents, corresponding with the material size provided by the manufacturer. After laser irradiation, the size of the material changed dramatically (the DLS graphs can be found in the Figure S1 in the Supplementary Materials). In the case of ethanol, the supernatant showed a size centered at 44 nm with an FWHM of 42 nm, while those of the sediment were 1474 nm and 1217 nm, respectively. The supernatant showed a great size reduction, but did not reach the range of ultra-small NPs. This could be caused by the presence of a matrix connecting the individual NPs, as has been observed in the case of Fe NPs synthesized by laser ablation in ethanol [22]. Furthermore, the sediment was mostly similar to the non-irradiated microparticles. In the case of water, the supernatant showed a size centered at 1.1 nm with an FWHM of 0.6 nm, while the sediment showed a bimodal size distribution, where one peak wsa centered at 511 nm with an FWHM of 437 nm, while the other peak was centered at 162 nm with an FWHM of 138 nm. Thus, the solid material in the supernatant showed sizes in the range of ultra-small NPs, while the sediment was, unlike in the case of ethanol, at least half the size of the precursor microparticles. In the case of ethylene glycol, the supernatant showed a size centered at 3.5 nm with an FWHM of 3.3 nm, while the sediment shows 1328 nm and 1314 nm, respectively. The use of ethylene glycol, therefore, led to a situation in between those of ethanol and water (i.e., it is possible to observe the iron ultra-small NPs, but the sediment still showed the presence of particles similar to the precursor). In the case of polyethylene glycol 400, the supernatant showed a size centered at 1.6 nm with an FWHM of 1.1 nm, while those of the sediment were 224 nm and 227 nm, respectively. Polyethylene glycol 400 represented the polar solvent for which it was possible to reach the maximum size reduction for the supernatant and the sediment. Finally, in the case of liquid nitrogen, the supernatant showed a bimodal size distribution, where one peak was centered at 5.3 nm with an FWHM of 2.2 nm, while the other was centered at 0.7 nm with an FWHM of 0.3 nm; the sediment showed a single size distribution, centered at 925 nm with an FWHM of 483 nm. As the DLS instrument’s detection limit (0.4 nm) was near the smallest peak, the peak around 0.7 nm was not reliable and probably represented an autocorrelation mistake in the instrument. The rest of the data suggested that photo-fragmentation also occurred at a low temperature (77 K). Given the employed laser parameters, the principal mechanism that led to size reduction of the microparticles was photothermal vaporization [40]. However, similar to the case of laser ablation in liquids, the current results demonstrate that more polar solvents benefit the production of smaller particles. After the microparticle vapor undergoes nucleation, the formed fragments interact with the surrounding liquid; meanwhile, as proposed by Gökce et al. [41], for the analogous ablation case, the solvents with higher polarity stabilize them and prevent their further growth more efficiently, thus facilitating the production of ultra-small NPs by LFL. The observation of large particles in all the sediments could be caused by partial microparticle evaporation and the non-homogeneous irradiation of the colloid particles. Moreover, it is essential to keep in mind that the DLS instrument provides information about the hydrodynamic size of particles, carrying information about the real particle diameters and the nearest surrounding molecules in the liquid. This should lead to the larger hydrodynamic sizes of the particles synthesized in EG and PEG, compared to the rest. However, the size was significantly increased only in the case of EG. In summary, TEM analysis must be performed to provide representative information about the real sizes of nanoparticles.

Figure 2, which shows the zeta potential of the different samples, indicates that stabilization leads to the preferential production of ultra-small NPs in solvents with a higher polarity and the colloidal stability of the subsequent product (|zeta potential| > 30 mV) [42]. After the laser irradiation, the sediment and supernatant samples corresponding to ethanol, water, and liquid nitrogen (particles dispersed in ethanol) displayed the lowest zeta potential value and, thus, the most deficient stability. For ethylene glycol, the zeta potential values were larger than those of the microparticles while, for polyethylene glycol 400, the zeta potential values were lower than in their microparticle counterparts, but still high enough to be considered stable. As has been extensively reported [38], a higher dipole moment of a solvent’s molecules leads to a stronger electric double layer and, consequently, a larger repulsion between particles. Therefore, as the dipole moments of these two solvents were significantly higher than those of the rest, they could enable the formation of more extensive electrical double layers, ensuring both the prevention of ultra-small NPs growth and long-term colloidal stability. Aside from the electrical double layer strength, the zeta potential value could also have been affected by other effects due to the experiment’s particular dynamics. As the colloids were continuously stirred, the collisions between them were increased, leading to subsequent agglomeration. This explains the high reductions in zeta potential value found for the rest of the samples. In the case of polyethylene glycol 400, being the largest molecule in the employed solvents and exhibiting the largest dipole moment, it may act as a quasi-static network, thus resisting the free movement of the ultra-small NPs by both electrostatic repulsion and the distance between them [43].

The TEM images displayed in Figure 3A, which were taken only for the supernatants that should include mostly ultra-small NPs or their agglomerates, agree with the DLS and zeta potential trends. In the images for water, ethylene glycol, and polyethylene glycol 400, particles with sizes below 3 nm are predominantly observed. This is because, when a solvent’s polarity is high, the more efficient it is in preventing the growth of particle fragments directly after their nucleation, thus boosting the preferential production of ultra-small NPs. In the case of water, it is additionally possible to observe larger bodies which seem to be surrounded by ultra-small NPs. As the reactivity of water with iron can promote more rapid oxidation in the ultra-small NPs than in their microparticle counterparts, the product’s interaction with water may result in further oxidation of possibly non-oxidized fragments. This leads to these large bodies, ultimately resulting in a detrimental effect on the sample’s long-term stability and zeta potential value.

For the ethanol sample, it is possible to see that the synthesized NPs were embedded in a matrix (see Figure S2, at a larger scale, in the Supplementary Material), which could be caused by the formation of iron oxide. In the nitrogen sample, the particles seemed to form large agglomerates (see also Figure S2 in the Supplementary Materials). Presumably, the low temperature of liquid nitrogen had a strong influence on the formation of Fe ultra-small NPs. The high-temperature gradient between the evaporate and the ultra-cold surrounding medium seemed to cause a rapid condensation of the vapor-phase iron nano-objects, therefore resulting in their rapid separation from the fragmented parts of the microparticle, finally leading to big bodies that seem to be frozen during the fragmentation process. Moreover, both supernatant samples that were dispersed in ethanol (i.e., samples created in ethanol and liquid nitrogen) displayed a broader particle size distribution than the rest. This may have been caused by the creation of the matrix in the case of the sample irradiated in ethanol, or by the weakening of the electrical double layer with the size reduction of particles, as inferred from their zeta potential values, increasing the probability of agglomeration.

Furthermore, the SAED diffractograms displayed in Figure 3B broaden this TEM analysis, by pointing out the presence of the tetragonal Fe**${}_{2}$**O**${}_{3}$** crystal (ICDD file: 65-390) or cubic Fe**${}_{3}$**O**${}_{4}$** crystal (ICDD file: 65-3107) in all of the samples, while only the ethanol sample displayed an additional crystal family that matched either with the hexagonal Fe crystal (ICDD files: 34-529) or with the monoclinic Fe${}_{5}$C${}_{2}$ crystal (ICDD file: 20-508). As has been highlighted in previous reports, water and organic solvents such as those employed in the current investigation can react with the incoming Fe NPs, leading to the formation of oxides or, as in the case of ethanol, promote the formation of carbides and nZVI (nano zero-valent iron), due to ethanol’s thermal decomposition [22,44,45]. However, the XPS data (shown in Figure 4) enabled the identification of three principal spectral ranges; furthermore, Fe 2p, O 1s, and C 1s provide a deeper understanding of this.

The ethanol sample exhibited two Fe 2p**${}_{3/2}$** peaks, the first at 706.9 eV and the second at 710.5 eV. The first one belongs either to nZVI or Fe${}_{5}$C${}_{2}$, as the complementary peak at 719.5 eV, associated with nZVI [46] or Fe${}_{5}$C${}_{2}$ [47] was also observed. The second observed Fe 2p**${}_{3/2}$** peak can be assigned to the presence of Fe**${}_{3}$**O**${}_{4}$** or Fe**${}_{2}$**O**${}_{3}$** nanostructures. The complementary Fe 2p**${}_{1/2}$** peak is situated at 724.2 eV [48], together with the Fe${}^{2+}$ peak belonging to Fe**${}_{3}$**O**${}_{4}$** or **${}_{2}$**O**${}_{3}$** at 712.2 eV and the Fe${}^{3+}$ belonging to Fe**${}_{3}$**O**${}_{4}$** at 726.5 eV. The complex spectrum of the ethanol sample suggests the formation of nZVI or iron carbide NPs embedded in an oxide matrix. However, in combination with the SAED analysis and EDX line scans (Figure S2B in the Supplementary Materials), the whole picture becomes more clear. As displayed in the corresponding SAED pattern, the sample showed crystalline families which can be assigned to both oxide types (i.e., Fe**${}_{2}$**O**${}_{3}$** and Fe**${}_{3}$**O**${}_{4}$**) and, as denoted by the EDX line scan, the Fe element signal significantly increased when scanning NPs embedded in the matrix, and a signal coming from C was also visible. Therefore, a more realistic picture is to consider that the synthesized structure includes nZVI, iron carbide, or iron oxide NPs embedded in an iron oxide matrix. As ethanol’s thermal decomposition—which leads to the formation of oxides—is usually attributed to the solvent’s interaction with hot iron atoms [22], the current results suggest that the iron vapor and the solvent in the gas state can interact directly in the cavitation bubble, leading to the formation of an iron oxide matrix with nZVI, iron carbide, or iron oxide NPs inside.

In the case of the sample synthesized in liquid nitrogen and further redispersed in ethanol, the peaks at 711.1 eV and 724.8 eV could be associated with Fe**${}_{2}$**O**${}_{3}$** or Fe**${}_{3}$**O**${}_{4}$**. However, unlike the case of ethanol, the Fe 2p satellites at 718.8 and 733.2 eV allowed for overcoming the ambiguity between both oxides. As such satellites are associated with the Fe${}^{3+}$ in Fe**${}_{2}$**O**${}_{3}$** structure, but also indicate the presence of a small amount of γ-Fe**${}_{2}$**O**${}_{3}$** [49], we finally assigned the peaks at 711.1 eV and 724.8 eV only to Fe**${}_{2}$**O**${}_{3}$**. Together with the SAED results, the current results indicate that synthesis in liquid nitrogen does not prevent the material’s oxidation, possibly mediated by the interaction between nucleating iron with the dissolved oxygen and the species resulting from the laser-dissociation of water molecules coming from the air.

The peaks exhibited by the sample synthesized in water showed a certain similarity to those found in the liquid nitrogen sample. It is possible to see the Fe 2p**${}_{3/2}$** peak at 710.5 eV, the Fe 2p**${}_{1/2}$** at 723.8 eV, the satellites of Fe**${}_{2}$**O**${}_{3}$** at 718.8 and 733.2 eV, and the peaks of Fe${}^{2+}$ belonging to Fe**${}_{3}$**O**${}_{4}$** at 712.1 eV and 725.6 eV. The combination of iron oxides may be due to the interaction between water in the gas state and the nucleating iron in the cavitation bubble or, alternatively, by their interaction with species coming from the laser-mediated molecular dissociation of water, which is rich in solvated electrons, hydroxyl radicals, and dissolved oxygen [50]. Unlike the case of liquid nitrogen, water is more viscous and, thus, can hold such species around the recently synthesized NPs, promoting the incomplete hydrogen reduction of the more thermodynamically stable Fe**${}_{2}$**O**${}_{3}$** to Fe**${}_{3}$**O**${}_{4}$** [51]. Finally, the lack of satellites for ethylene glycol points only to the presence of Fe**${}_{3}$**O**${}_{4}$**. Although the graph included the Fe 2p**${}_{3/2}$** peak at 710.6 eV, and a Fe 2p**${}_{1/2}$** at 723.9 eV, which can be associated with both, Fe**${}_{2}$**O**${}_{3}$** or Fe**${}_{3}$**O**${}_{4}$**, the peaks of the Fe${}^{3+}$ which belong just to Fe**${}_{3}$**O**${}_{4}$** at 713.9 eV and 726.7 eV helped to address this ambiguity [48,49]. Similar to the case of water, the laser radiation could promote the molecular dissociation of ethylene glycol, which is also rich in solvated electrons, hydroxyl radicals, and dissolved oxygen [52]. However, unlike that case, ethylene glycol is more viscous and, therefore, can hold the species due to molecular dissociation around the nucleating Fe more effectively, leading to the preferential formation of Fe**${}_{3}$**O**${}_{4}$** nanostructures.

The O 1s and C 1s spectra analysis showed multiple types of organic residuals which were ascribable to the solvents found in the ethylene glycol sample, with ethanol, water, and liquid nitrogen environments being associated with lower amounts of contamination, typically due to adventitious carbon. In the case of ethanol and ethylene glycol, the adsorbed hydroxyl groups (C–OH) or adsorbed non-lattice oxygens were indicated by peaks at 533.7–533.8 eV in the O 1s spectra and, for the rest of the samples, at 285.8–286.4 eV in C 1s spectra. Carbonyl (C=O) groups were also revealed by the presence of a peak at 532.5 eV in the ethylene glycol sample spectrum and at 288.5–288.8 eV in the C 1s spectra of the rest of the samples. Moreover, it was possible to identify the presence of carboxyl groups (O=C–O), which were perceived as two connected groups (C=O and C–OH) indicated by the peaks at 531.0–531.6 eV in all C 1s spectra and at 289.3 eV in the O 1s spectrum for the ethylene glycol sample [49,53,54].

As the identified bonds represent information about the particle–liquid interface composition and particle lattice content, the O 1s and C 1s spectra may provide information about the stabilization of iron ultra-small NPs. In the case of ethanol, the Fe 2p, together with the TEM results, indicated that an iron matrix containing nZVI, iron carbide, or iron oxide NPs was formed. However, the lowest relative intensity difference between the lattice oxygen peaks and O–C=O and C–OH bonds in the O 1s spectrum, accompanied by the largest one from the C–C bond and C=O bond in the C 1s spectrum, in comparison to the rest of the samples, indicated that the stabilization followed a two-step mechanism. On one hand, the iron oxide structure formed around the NPs, due to the interaction between the solvent and the iron in the vapor state, might prevent further particle growth, acting as the first stabilization step in this way. The second step can be connected to its interaction with the rest of the solvent’s molecules, the poor ability of which to form a strong electrical double layer around the incoming products led to the low zeta potential values observed. In the case of water, as for the rest of the samples, the hydroxyl groups and oxidation seemed to be the principal cause of particle stabilization. However, as revealed by the zeta potential, its long-term colloidal stability seemed to be affected by the high reactivity between water and partially oxidized ultra-small iron NPs, which could undergo further oxidation outside the cavitation bubble. For liquid nitrogen, it showed similar O 1s and C 1s spectra to those of water, indicating that water from the air during the photo-fragmentation could provide changes in the chemical composition of particles and contribute to their stabilization, together with the rapid condensation of vapor. However, the low polarity of ethanol where the incoming particles were redispersed led to poor long-term stability. In the case of ethylene glycol, which showed the largest concentration of carboxylic groups, as well as O 1s and C 1s spectra that appeared to be a combination between that of pure ethylene glycol [55] and the one observed when water and species from water molecular’s dissociation were involved in the nucleation of particles. It was considered that the XPS spectra revealed a lack of detachment of molecules from the surface of the ultra-small iron NPs after the drying process. This indicates that the solvent firmly attaches to the particle’s surface; moreover, as the binding groups were the same as in the rest of the solvents, such adherence was then attributed to this solvent’s high polarity, which also enabled the long-term stability of the particles. The EDX line scans (Figure S2B in the Supplementary Materials) of the samples supported the XPS data, confirming that the particles were effectively composed of iron. Moreover, the prominent signal of oxygen, compared to iron, in ethylene glycol indicated the presence of liquid molecules while taking the EDX spectra of the produced ultra-small NPs. As the spectra were obtained for the ethylene glycol dried sample in a vacuum of ∼10${}^{-4}$ Pa, these measurements confirm the strong adherence of the molecules over the recently synthesized particles.

Note that it was not possible to perform XPS, SAED, and EDX line scan measurements of the sample synthesized in polyethylene glycol 400, due to the impossibility of removing the solvent without modifying the composition of the synthesized NPs (e.g., by heating), due to the extremely low volatility of this liquid, or by lyophilization. Moreover, even when it was possible to take clear TEM micrographs, the wet sample’s prolonged exposure to the electron beam, as required for SAED and EDX line scanning, promoted the burning of the surrounding polyethylene glycol 400 molecules, thus making their correct analysis impossible.

After analyzing the DLS, zeta potential, TEM, and XPS data, it became evident that the selection of solvent has a substantial impact on the synthesis of Fe ultra-small NPs, leading to different sizes, stability, and surface nature. However, while considering crossing the line between basic research and applied science, it is fundamental to determine the influence of these solvents on the amount of material that can be produced. For this, we measured the concentration of Fe in the different sample supernatants by ICP-OES. The water and nitrogen samples showed Fe concentrations of less than 0.02 mg/L, while ethanol, ethylene glycol, and polyethylene glycol 400 showed concentrations of 6.35 mg/L, 2.47 mg/L, and 1.19 mg/L, respectively (the ICP-OES details can be found in the Figure S3 in the Supplementary Materials). Even though the sample in ethanol showed the largest Fe concentration, it should be kept in mind that it permits the particle’s size to grow over time. Therefore, the liquids that provided the best ultra-small Fe NPs production rate were those with the highest polarity (i.e., ethylene glycol and polyethylene glycol 400).

In summary, study of the particle size modification, chemical structure, productivity, and colloidal stability enabled us to determine that the concept of employing polar solvents to produce ligand-free NPs, which has already been proposed for laser ablation in liquids, can be extended to the production of ligand-free ultra-small NPs when using laser fragmentation in liquids. In particular, these findings indicate that, when photothermal evaporation drives the fragmentation process of iron, the interaction between the particle evaporate and the polar molecules and species coming from their dissociation promote the structural modification of iron, constituting the first stabilization mechanism which impacts on the technique’s productivity. Once the ultra-small NPs interact with the liquid medium, thus forming a colloid, the solvents with a higher polarity provide a stronger electrical double layer, ensuring the possibility to maintain the ultra-small size for extended periods without the necessity of requiring further stabilization ligands. The current study culminated in the evidence that solvents with a high dipolar moment, such as ethylene glycol or polyethylene glycol 400, possess the optimal parameters for producing highly stable colloids composed of the largest amount of ligand-free ultra-small iron NPs. Furthermore, the phase composition, which is controlled by the solvent, is promising for future advances in the design of crystallographic precise iron building blocks for applications in biomedicine, catalysis, or material science. In particular, Fe**${}_{3}$**O**${}_{4}$** and ligand-free ultra-small NPs produced by the employment of ethylene glycol may be of great value, due to their growing use as MRI contrast agents and in theranostics. Moreover, future advances in theoretical modeling will enable a better understanding of the laser-mediated crystallization processes and the extrapolation of the current findings to multiple liquids, thus boosting this method’s prospects to penetrate more knowledge disciplines.

## Figures and Tables

**Figure 1:**
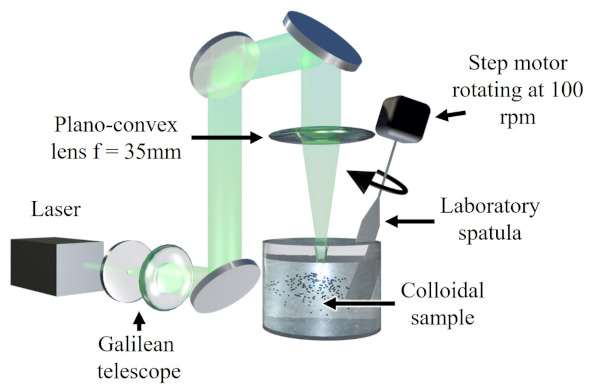
Experimental setup used for the synthesis of Fe ultra-small NPs by the photothermal vaporization of carbonyl iron microparticles.

**Figure 2:**
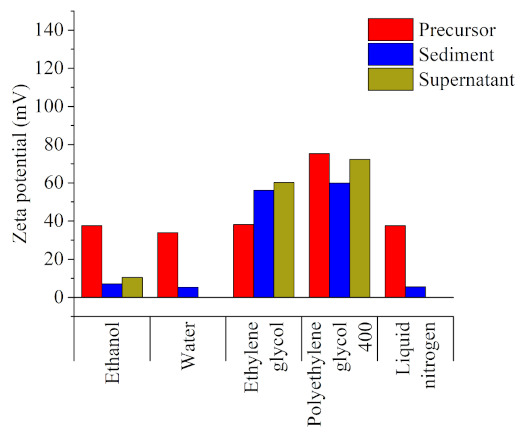
Zeta potential values for the precursor (the non-irradiated colloid of the carbonyl iron microparticles), the sediment, and the supernatant extracted from the irradiated samples, all dispersed in the corresponding solvents (ethanol was used in case of the samples prepared in liquid nitrogen).

**Figure 3:**
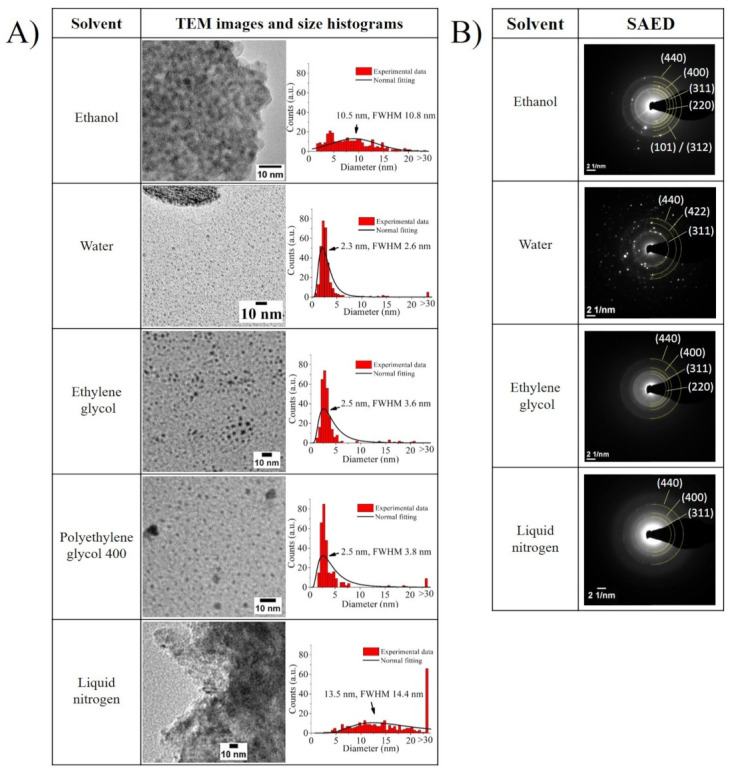
(**A**) Representative TEM images and size histogram of the different samples (obtained by counting 300 particles per each sample). The average size calculated from the normal fitting is indicated in the histograms. (**B**) SAED images of all samples (aside from polyethylene glycol 400). The d-spacings were assigned to the crystallographic families indicated in the Table S1 in the Supplementary Materials.

**Figure 4:**
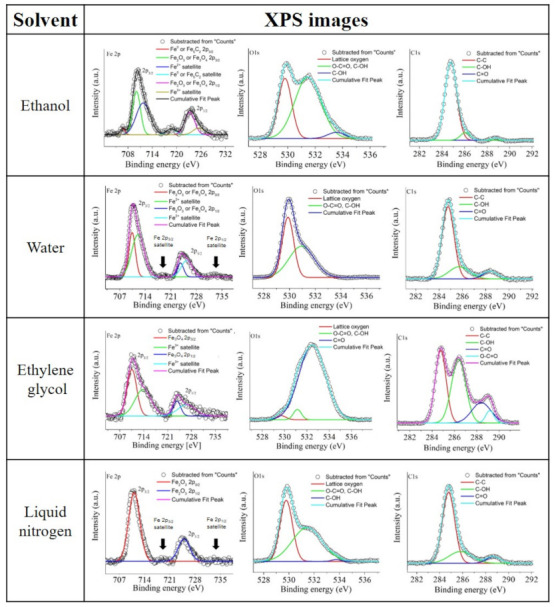
List of XPS spectra for the supernatant of each sample. The binding energies were corrected, taking the C–C peak of each sample as a reference.

## Supplementary Materials

The following are available at https://www.mdpi.com/article/10.3390/nano11061538/s1. The supplementary material includes the calculations employed to obtain the laser fluence and full evaporation threshold, the DLS graphs of supernatant and sediment for all the samples, a table with the SAED diffraction data displaying the d-spacing values and appropriate crystallographic families, a figure displaying the TEM images of all the samples (at a larger scale than those reported in the main text), the EDX line scans, and raw ICP-OES data.
