# Supplementary material for: On the Use of Laser Fragmentation for the Synthesis of Ligand-Free Ultra-Small Iron Nanoparticles in Various Liquid Environments
Source: Nanomaterials (Basel). 2021 Jun 10;11(6):1538. doi: 10.3390/nano11061538 (PMC8230550; doi:10.3390/nano11061538)
Supplement: Supplementary file 1 [file nanomaterials-11-01538-s001.zip › nanomaterials-1224732-SI.pdf]

# Supplementary Materials: On the Laser Fragmentation for the Synthesis of Ligand-Free Ultra-Small Iron Nanoparticles in Various Liquid Environments

**Ondřej Havelka [1], Martin Cvek [2], Michal Urbánek [2], Dariusz Łukowiec [3], Darina Jašíková [1], Michal Kotek [1], Miroslav Černík [1], Vincenzo Amendola[3] and Rafael Torres-Mendieta [1,*]**

- Estimation of laser fluence, roughly considering a beam's free propagation, and calculation of the full evaporation fluence threshold.
- Supporting DLS graphs of supernatant and sediment for all the samples.
- SAED diffraction data displaying the crystallographic families corresponding to the specific type of Fe-based crystals.
- Supporting figure displaying the TEM images of all the samples on a bigger scale than the one reported in the main text, and supporting EDX line scanning graphs of the individual nanoparticles in different solvents.
- The raw ICP-OES data confirming the presence of iron.

The calculation of the laser fluence was determined according to the following set of standard formulas:

$$P = 7.0\, W\, (measured\, average\, power) \tag{1}$$

$$N = 2000\, (number\, of\, pulses) \tag{2}$$

$$E = \frac{P}{N}\, (energy\, per\, pulse) \tag{3}$$

$$E = 3.5\, mJ \tag{4}$$

$$f = 35\, mm\, (local\, length\, of\, plano - convex\, focusing\, lens) \tag{5}$$

$$s = 12\, mm\, (measured\, diameter\, of\, unfocused\, beam) \tag{6}$$

$$\lambda = 527\, nm\, (laser's\, central\, wavelength) \tag{7}$$

$$d = \frac{2 \cdot 1.22 \cdot \lambda \cdot \frac{f}{s}}{\sqrt{2 \cdot ln(2)}}\, (diameter\, of\, focused\, beam) \tag{8}$$

$$d = 3.19 \cdot 10^{-4}\, cm \tag{9}$$

$$S = \pi \cdot \left(\frac{d}{2}\right)^2\, (area\, of\, focused\, laser\, beam) \tag{10}$$

$$S = 8 \cdot 10^{-8}\, cm^2 \tag{11}$$

$$J = \frac{E}{S}\,(laser\,fluence) \tag{12}$$

$$J = 4.3 \cdot 10^4\, J/cm^2 \tag{13}$$

The calculation of the fluence threshold for heating-melting-evaporation model was determined according to the following set of formulas:

$$E_{abs} = \frac{E_0}{S_0} \cdot \sigma_{abs} = \Psi \cdot \sigma_{abs} \tag{14}$$

where $E_0$ represents the energy of a single pulse, $S_0$ the laser beam cross-section (the area of light that is perpendicular to the beam direction), $\Psi = E_0/S_0$ the laser fluence and $\sigma_{abs}$ the particle absorption cross-section, which describes the energy absorbed by the irradiated particle.

$$\Psi \cdot \sigma_{abs} = m_p \cdot \int_{T_0}^{T} c_p(T) \cdot dT \tag{15}$$

where $m_p = \rho_p \pi d_p^3/6$ is the particle mass, $\rho_p$ is the density of the particle, $d_p$ is the diameter of the particle and $T_0$ is the initial temperature of the particle and T is the final temperature of the particle.

$$\Psi \cdot \sigma_{abs} = m_p \cdot \int_{T_0}^{T_m} c_p^s(T) \cdot dT + m_p \cdot \Delta H_m + m_p \cdot \int_{T_m}^{T_b} c_p^l(T) \cdot dT + m_p \cdot \Delta H_{ev} \tag{16}$$

Here, $c_p^s$ represents the heat capacity of the material in the solid state, $c_p^l$ the heat capacity in the liquid state, $T_m$ the melting temperature, $T_b$ the boiling temperature and as previously mentioned, $\Delta H_m$ the enthalpy of the melting and $\Delta H_{ev}$ the enthalpy of the evaporation.

$$\Psi \cdot \sigma_{abs} = m_p \cdot [(H_{T_m} - H_{T_0}) + \Delta H_m + (H_{T_b} - H_{T_m}) + \Delta H_{ev}] \tag{17}$$

where $H_{T_m} - H_{T_0}$ represents the relative enthalpy to reach the melting point and $H_{T_b} - H_{T_m}$ the relative enthalpy to reach the boiling point.

$$\sigma_{abs}(d_p) \cdot \mathbf{d}(\Psi) = \Delta H_{ev} \cdot \mathbf{d}(m_p) \tag{18}$$

which after the substitution of $m_p$ and the editing takes the following form

$$\mathbf{d}(\Psi) = \frac{\pi}{2} \cdot \rho_p \cdot \Delta H_{ev} \cdot \frac{d_p^2 \cdot \mathbf{d}(d_p)}{\sigma_{abs}(d_p)} \tag{19}$$

Then, the final equation that enables to calculate the required fluence to fully evaporate micro particles will be

$$\begin{aligned} \Psi \;=\; & \frac{\pi \cdot d_p^3 \cdot \rho_p}{6 \cdot \sigma_{abs}} \cdot [(H_{T_m} - H_{T_0}) + \Delta H_m + (H_{T_b} - H_{T_m})] + \\ & \frac{\pi \cdot \rho_p}{2} \cdot \Delta H_{ev} \cdot \left( \int_{d_p}^{0} \frac{d_p^2}{\sigma_{abs}(d_p)} \cdot \mathbf{d}(d_p) \right) \end{aligned} \tag{20}$$

Note, the particle cross-section was calculated by Mie-theory calculator [1] and the required values of the quantities were taken from the database [2].

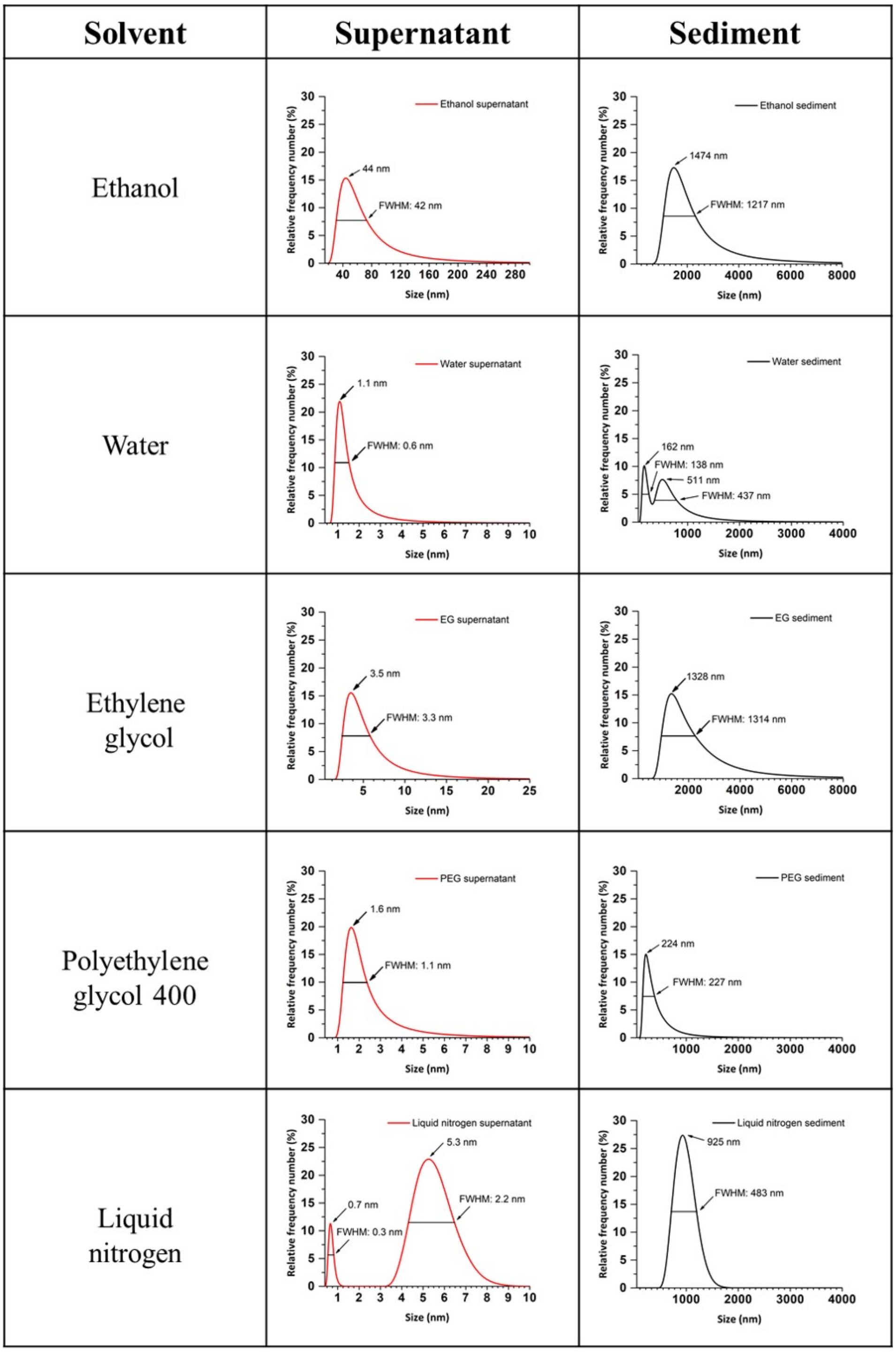


**Figure S.1.** Representative DLS images of sediment and supernatant samples.

**Table S1.** SAED parameters of samples corresponding to the specific crystallographic families in Fe-based crystals. Tetragonal $Fe_2O_3$ crystal (ICDD file: 65-390), cubic $Fe_3O_4$ crystal (ICDD file: 65-3107), hexagonal Fe crystal (ICDD files: 34-529), and the monoclinic $Fe_5C_2$ crystal (ICDD file: 20-508)

| **Ethanol** | | | | **Crystal type** | **Water** | | | | **Crystal type** |
|---|---|---|---|---|---|---|---|---|---|
| **No** | **1/2r ($nm^{-1}$)** | **d-spacing (A)** | **hkl** | | **No** | **1/2r ($nm^{-1}$)** | **d-spacing (A)** | **hkl** | |
| 1 | 6.741 | 2.967 | (220) | $Fe_2O_3$ / $Fe_3O_4$ | 1 | 7.830 | 2.554 | (311) | $Fe_2O_3$ / $Fe_3O_4$ |
| 2 | 8.001 | 2.500 | (311) | $Fe_2O_3$ / $Fe_3O_4$ | 2 | 11.719 | 1.707 | (422) | $Fe_2O_3$ / $Fe_3O_4$ |
| 3 | 9.544 | 2.096 | (400) | $Fe_2O_3$ / $Fe_3O_4$ | 3 | 13.780 | 1.451 | (440) | $Fe_2O_3$ / $Fe_3O_4$ |
| 4 | 10.970 | 1.823 | (101) / (312) | Fe / $Fe_5C_2$ | 4 | | | | |
| 5 | 13.455 | 1.486 | (440) | $Fe_2O_3$ / $Fe_3O_4$ | 5 | | | | |
| **Ethylene glycol** | | | | **Crystal type** | **Liquid nitrogen** | | | | **Crystal type** |
| **No** | **1/2r ($nm^{-1}$)** | **d-spacing (A)** | **hkl** | | **No** | **1/2r ($nm^{-1}$)** | **d-spacing (A)** | **hkl** | |
| 1 | 6.816 | 2.934 | (220) | $Fe_2O_3$ / $Fe_3O_4$ | 1 | 7.957 | 2.514 | (311) | $Fe_2O_3$ / $Fe_3O_4$ |
| 2 | 7.966 | 2.511 | (311) | $Fe_2O_3$ / $Fe_3O_4$ | 2 | 9.652 | 2.072 | (400) | $Fe_2O_3$ / $Fe_3O_4$ |
| 3 | 9.511 | 2.103 | (400) | $Fe_2O_3$ / $Fe_3O_4$ | 3 | 13.608 | 1.470 | (101) | $Fe_2O_3$ / $Fe_3O_4$ |
| 4 | 13.597 | 1.471 | (440) | $Fe_2O_3$ / $Fe_3O_4$ | 4 | | | | |

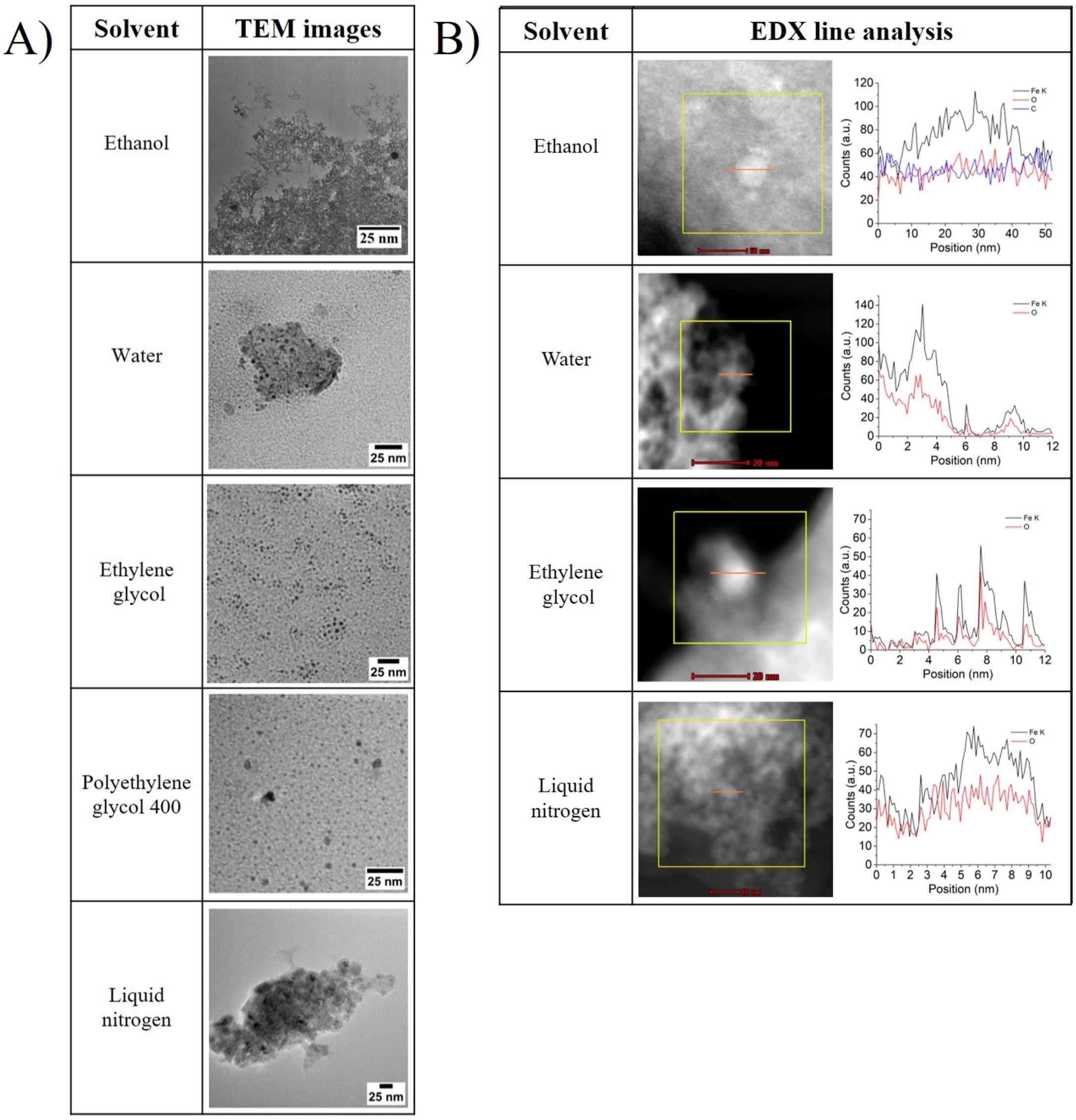


**Figure S.2.** A) Representative TEM images of the different samples where the bar scale is 25 nm. In these images, it is possible to appreciate the oxide matrices containing the iron-based particles synthesized in ethanol, and the agglomerates of the sample synthesized in liquid nitrogen and further redispersed in ethanol. B) Representative EDX line scans of the individual ultra-small NPs found in the samples' supernatant.

[1] https://omlc.org/calc/mie_calc.html [online 21-04-2021]
[2] https://www.webelements.com/iron/thermochemistry.html [online 25-04-2021]

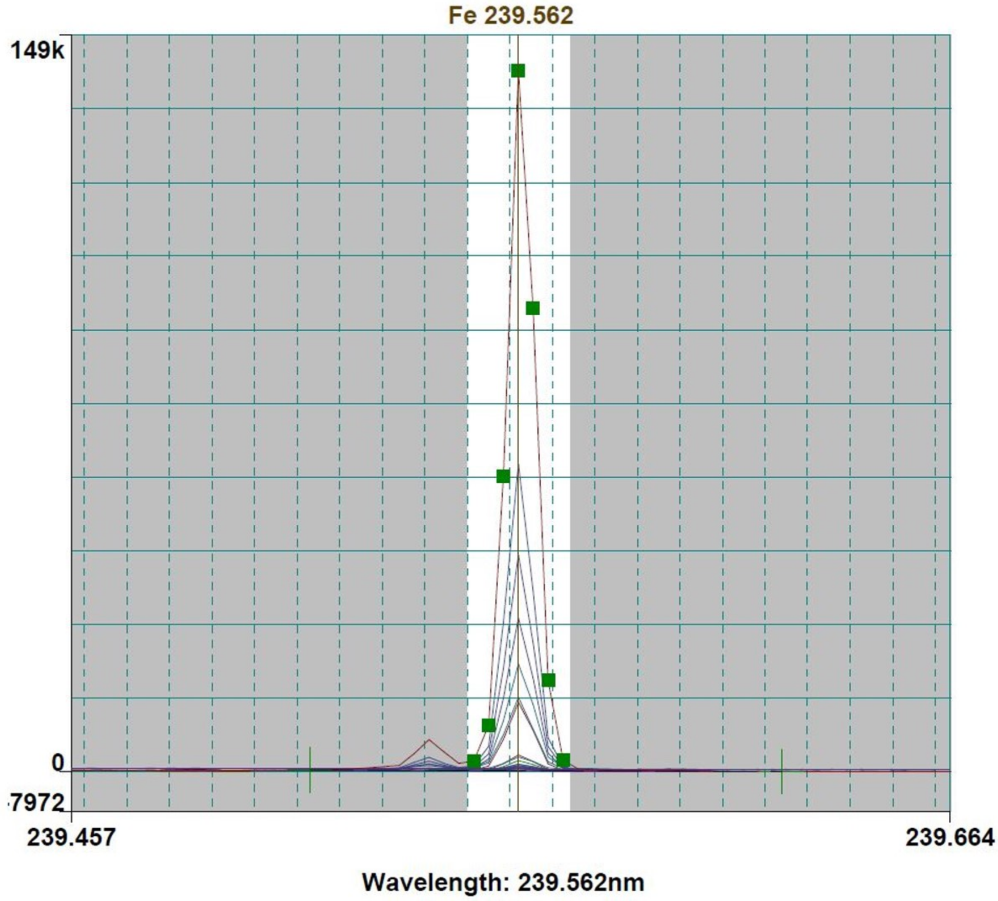


**Figure S.3.** Details from ICP-OES measurements. The main iron peak was measured at 239.56 nm, and is possible to observe smaller peak at 239.54 nm, also belonging to the iron.
